# PCV2 and PRV Coinfection Induces Endoplasmic Reticulum Stress via PERK-eIF2α-ATF4-CHOP and IRE1-XBP1-EDEM Pathways

**DOI:** 10.3390/ijms23094479

**Published:** 2022-04-19

**Authors:** Si Chen, Xue Li, Xinwei Zhang, Guyu Niu, Lin Yang, Weilong Ji, Liying Zhang, Linzhu Ren

**Affiliations:** College of Animal Sciences, Key Lab for Zoonoses Research, Ministry of Education, Jilin University, 5333 Xi’an Road, Changchun 130062, China; sichen20@mails.jlu.edu.cn (S.C.); lixue9915@mails.jlu.edu.cn (X.L.); xwzhang17@mails.jlu.edu.cn (X.Z.); niugy9916@mails.jlu.edu.cn (G.N.); linyang20@mails.jlu.edu.cn (L.Y.); jiwl19@mails.jlu.edu.cn (W.J.)

**Keywords:** porcine circovirus type 2, porcine pseudorabies virus, coinfection, endoplasmic reticulum stress (ERS), transcriptome sequencing

## Abstract

Porcine circovirus 2 (PCV2) and pseudorabies virus (PRV) are two important pathogens in the pig industry. PCV2 or PRV infection can induce endoplasmic reticulum stress (ERS) and unfolded protein response (UPR). However, the effect of PCV2 and PRV coinfection on the ERS and UPR pathways remains unclear. In this study, we found that PRV inhibited the proliferation of PCV2 mainly at 36 to 72 hpi, while PCV2 enhanced the proliferation of PRV in the middle stage of the infection. Notably, PRV is the main factor during coinfection. The results of the transcriptomic analysis showed that coinfection with PCV2 and PRV activated cellular ERS, and upregulated expressions of the ERS pathway-related proteins, including GRP78, eIF2α, and ATF4. Further research indicated that PRV played a dominant role in the sequential infection and coinfection of PCV2 and PRV. PCV2 and PRV coinfection induced the ERS activation via the PERK-eIF2α-ATF4-CHOP axis and IRE1-XBP1-EDEM pathway, and thus may enhance cell apoptosis and exacerbate the diseases.

## 1. Introduction

Porcine circovirus type 2 (PCV2) is one type of porcine circovirus, which belongs to the *Circoviridae* family *Circovirus* genus, containing a single-stranded, positive-sense, and circular DNA in the virion [1,2]. PCV2 is considered the causative pathogen of porcine circovirus diseases and porcine circovirus-associated diseases (PCVD/PCVAD), including post-weaning multisystemic wasting syndrome (PMWS), porcine dermatitis and nephropathy syndrome (PDNS), porcine respiratory disease complex (PRDC), and reproductive failure [1,2]. The virus infection has been reported in swine farms all over the world, with a high positive rate even exceeding 90%, and has caused great losses in the swine industry [1,2]. Notably, PCV2 infection causes subclinical symptoms, but it can also lead to immunosuppression, which provides a better condition for coinfection and/or the secondary infection of other pathogens [1,3,4,5,6,7,8]. Furthermore, PCV2 infection can weaken the stimulation of the vaccine to the immune system and reduce the protective effect of the vaccine, or increase the virulence of the modified live vaccine [1,9]. Therefore, it is of great significance for the prevention and treatment of these clinical diseases to understand the situation and pathogenesis of concurrent infection.

Among the pathogens co-infected or sequentially infected with PCV2, viruses are the most important and should not be ignored, as they usually cause acute and serious diseases after co- or sequential infection [1,9,10]. As reported, the coinfection rates of PCV2 with porcine reproductive and respiratory syndrome virus (PRRSV), pseudorabies virus (*Suid herpesvirus* 1 or PRV), classical swine fever virus (CSFV), and porcine epidemic diarrhea virus (PEDV) were 26.73%, 18.37%, 13.06%, and 3.47%, respectively, in Shandong province in China from 2015 to 2018 [11]. We recently evaluated samples isolated in 2018–2021 from Jilin province, China, and found that coinfection rates of PCV2 and PRV ranged from 1.19% to 11.93%. 

PRV belongs to the genus *Varicellovirus*, *Alphaherpesvirinae* subfamily, and the family *Herpesviridae*. PRV infection can lead to nervous system disorder with high mortality in piglets, growth retardation and respiratory diseases in growing pigs, and reproductive failure in sows [11,12,13], and is one of the important diseases threatening the pig industry at present. It is worth noting that PRV can not only infect swine and wild boar but also can infect several mammals, including humans, dogs, and rodents [11,13,14]. 

It was reported that PCV2 can induce mitochondrial apoptosis, endoplasmic reticulum stress (ERS), unfolded protein response (UPR), and autophagy to promote viral replication via protein kinase R (PKR)-like ER kinase (PERK) pathway [15,16,17,18,19]. Meanwhile, PRV activates PERK and inhibits stress granules formations via dephosphorylating eukaryotic initiation factor 2α (eIF2α) [20]. The dephosphorylation of eIF2α was promoted by upregulation of growth arrest and DNA damage-inducible protein 34 (GADD34) during PRV infection [21]. PRV infection also induced ERS and activated the inositol requiring enzyme 1 (IRE1), X-box-binding protein 1(XBP1), eIF2α, and activating transcription factor 4 (ATF4) pathways [22]. However, there is no report on the ERS and UPR pathways caused by PCV2 and PRV coinfection.

In this study, porcine kidney epithelial cells (PK-15) infected with PCV2 and PRV alone, sequentially, or jointly for 12 h were analyzed by transcriptomic analysis, followed by evaluation of the differentially expressed genes (DEGs) and related ERS pathways involved in virus infection, which was helpful to further clarify the pathogenic mechanism of PCV2 and PRV coinfection.

## 2. Material and Methods

### 2.1. Cells and Viruses

Porcine kidney epithelial cells (PK-15) were cultured in Dulbecco’s Modified Eagle Medium (DMEM, Thermo Fisher Scientific, Shanghai, China) supplemented with 5% fetal bovine serum (FBS, Clark, Bioscience, Shanghai, China), and maintained in a humidified incubator at 39 °C and 5% CO_2_. 

The virulent wild-type PRV strain [23] and PCV2 strain CC1 [24] were used in this study. 

### 2.2. Virus Infection

PK-15 cells (7 × 10^5^ cells/well) were cultured in 12-well plates to reach 75% confluence. Then, cells were washed with phosphate buffer saline (PBS) three times and divided into 6 groups for virus infection.

Group 1 was used as a control group (PK-15 group). Group 2 was infected with PCV2 alone (PCV2 group), and group 3 was infected with PRV alone (PRV group). Cells were infected with PCV2 (multiplicity of infection, MOI = 0.5) or PRV (MOI = 0.01) alone for adsorption for 1 h. Then, cells were washed with PBS three times and then maintained in DMEM supplemented with 5% FBS for the indicated time, followed by evaluation via PCR, real-time PCR, or Western blotting.

Group 4 and group 5 were two sequentially infected groups. In Group 4 (PCV2-12h-PRV group), cells were infected with PCV2 for 1 h, washed with PBS three times, and cultured in DMEM supplemented with 5% FBS. Then, 12 h later, cells were incubated with PRV for 1 h and washed with PBS three times, and cultured in DMEM supplemented with 5% FBS for the indicated time. In Group 5 (PRV-12h-PCV2 group), cells were infected with PRV for 1 h, washed with PBS three times and cultured in DMEM supplemented with 5% FBS. Then, 12 h later, cells were incubated with PCV2 for 1 h and washed with PBS three times and cultured in DMEM supplemented with 5% FBS for the indicated time. Then, samples were collected and evaluated via real-time PCR or Western blotting.

Group 6 (PCV2+PRV group) was co-infected with PCV2 and PRV for 1 h. Then, cells were washed with PBS three times and cultured in DMEM supplemented with 5% FBS for the indicated time. Samples were collected and evaluated via PCR, real-time PCR, or Western blotting.

### 2.3. RNA Extraction

Cells of different groups were collected at 4-, 8-, 12-, 24-, 36-, and 48-h post-infection (hpi). Total RNA was extracted by the TRNzol Universal Reagent (Tiangen, Beijing, China) according to the manufacturer’s instructions. The concentration and purity of total RNA were assessed by Nanodrop 2000 (Thermo Fisher Scientific, Shanghai, China). 

### 2.4. Real-Time Quantitative PCR (RT-qPCR)

Reverse transcription was conducted with the Fastking gDNA Dispelling RT SuperMix (Tiangen, Beijing, China) according to the manufacturer’s instructions.

RT-qPCR was performed using 2×SYBR Green qPCR Master Mix (Bimake, Houston, TX, USA). The PCR program was as follows: 95 °C for 300 s, followed by 40 cycles of 95 °C for 15 s, 57 °C for 30 s, and 72 °C for 30 s, followed by melting curve analysis. Relative expression levels were calculated by using the 2^−ΔΔ^Ct method using GAPDH as the reference gene. The primer sequences are listed in Supplemental Table S1.

### 2.5. One-Step Growth Curve

The cells and supernatant of six groups were collected at 12-, 24-, 36-, 48-, 60-, and 72-h post-infection (hpi), followed by viral genomic DNA extraction using TIANamp Virus DNA/RNA Kit (Tiangen, Beijing, China). Viral DNA was determined by RT-qPCR and growth curves were generated based on the viral copies at the indicated time.

### 2.6. RNA-Seq Analysis

Total RNA was extracted by the TRNzol Universal Reagent (Tiangen, Beijing, China) from four groups mentioned above (PK-15, PCV2, PRV, and PCV2+PRV) at 12 hpi, and quantified using NanoDrop and Agilent 2100 bioanalyzer (Thermo Fisher Scientific, Shanghai, China). Then, the total RNA was analyzed for transcriptome sequencing analysis by BGI Technology Co., Ltd. (Shenzhen, China). The library preparation and RNA-Seq were carried out by the DNBSEQ^TM^ Sequencing platform.

### 2.7. Gene Ontology (GO) and Kyoto Encyclopedia of Genes and Genomes (KEGG) Enrichment Analysis

GO functional enrichment and KEGG pathway enrichment of DEGs were performed and the enriched genes were further classified with a Q value < 0.05 according to the protocols described by the Dr. TOM system (BGI Genomics, Shenzhen, China). Volcano plot, heatmap, and Venn diagram of DEGs between the control group (PK-15) and infected groups were analyzed by the Dr. TOM system.

### 2.8. Protein–Protein Interaction Network Analysis 

Biological networks analysis of DEGs was performed via the Dr. TOM system, using key driver analysis (KDA), and protein–protein interaction network (PPI network). Terms and pathways with *p* < 0.05 were significantly enriched. 

### 2.9. Western Blotting

Cells collected from each group at the indicated time points (4, 8, 12, 24, 36, and 48 hpi) were lysed on ice with cell lysis buffer (Beyotime, Shanghai, China) containing protease inhibitor (100×, BOSTER, Wuhan, China) and phosphatase inhibitor (50×, Beyotime, Shanghai, China). After centrifugation at 12,000 rpm for 15 min at 4 °C, the supernatant was collected and quantified with a BCA protein assay kit (Beyotime, Shanghai, China).

The protein solution was mixed with 5× SDS-PAGE loading buffer and boiled for 10 min, followed by separation on 12% SDS-PAGE gel at 80 V for 3 h. Subsequently, proteins were transferred to polyvinylidene difluoride (PVDF) membranes (Millipore, Merck, Darmstadt, Germany) at 300 mA for 60 min at 4 °C. The membranes were blocked in 5% skimmed milk (diluted with Tris-buffered saline containing 0.1% Tween 20, TBST) at room temperature for 90 min, and then incubated with the indicated primary antibodies dissolved in TBST overnight at 4 °C or room temperature for 2 h. After washing with TBST three times, the membrane was incubated with secondary antibodies HRP-conjugated goat anti-rabbit IgG (Wanleibio, Shenyang, China, 1:5000) or HRP-conjugated goat anti-mouse IgG (Beyotime, Shanghai, China, 1:1000) at room temperature for 1 h. The signal was developed using the ECL reagent (Mei5bio, Beijing, China), and examined via Tanon 5200 system (Bioanalytical Imaging System, Azure Biosystems, Dublin, CA, USA). The mean densities of the protein bands were measured using ImageJ software. β-actin was used as an internal control.

### 2.10. XBP1 Splicing Analysis 

Total RNA was extracted at the indicated time points (4, 8, 12, 24, 36, and 48 hpi) from cells using TRNzol Universal Reagent (Tiangen, Beijing, China). RNA was reverse transcribed using Fastking gDNA Dispelling RT SuperMix (Tiangen, Beijing, China), and PCR was performed with 2 × *Taq* PCR MasterMix II (Tiangen, Beijing, China) using the primers listed in Supplemental Table S1. PCR reactions were 94 °C for 3 min, 35 cycles of 94 °C for 30 s, 58 °C for 30 s, and 72 °C for 20 s, followed by 72 °C for 5 min. Amplicons were separated on 2% agarose gel and examined using the Alphaimager EC imaging system (Alpha Innotech, San Jose, CA, USA).

### 2.11. Statistical Analysis

Two-way ANOVA statistical analysis was performed using GraphPad 7.0 (GraphPad Software, San Diego, CA, USA). A *p*-value < 0.05 was considered statistically significant. Each value was the mean ± standard deviation (SD) of three replicates.

## 3. Results

### 3.1. Growth Kinetics of PCV2 and PRV in Different Combinations of Infection Groups in PK-15 Cells

**Figure 1:**
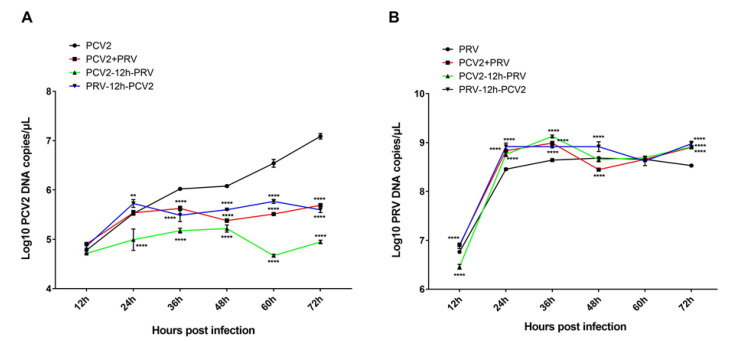
Growth curves of PCV2 (**A**) and PRV (**B**) in different infection groups. PK-15 cells were infected with different combinations of PCV2 and/or PRV for 12, 24, 36, 48, 60, and 72 h, followed by evaluation using real-time PCR. **, *p*-value < 0.01; ****, *p*-value < 0.0001.

PK-15 cells were single- (PCV2 or PRV group), sequential- (groups PCV2-12h-PRV and PRV-12h-PCV2), or co-infected (PCV2+PRV group) with PCV2 and/or PRV for 12, 24, 36, 48, 60, and 72 h, followed by evaluation of the virus replication in the cells. The results of growth curves showed that the copies of PCV2 were decreased in the sequential- and co-infected groups compared with that of the PCV2 infection alone (Figure 1A), suggesting that PRV infection inhibits the proliferation of PCV2. The most obvious period of inhibition was at 36 to 72 hpi. Furthermore, the growth curves of Figure 1B showed that the copies of PRV were increased in the sequential- and co-infected groups compared with that of the PRV alone group at 24 to 36 hpi (Figure 1B), suggesting that PCV2 infection enhances the proliferation of PRV, especially in the middle stage of the infection. In addition, the influence of the coinfection group on virus infection was similar to that of sequential infection groups.

### 3.2. The Influence of the Coinfection Group on Cell Viability Was Similar to That of Sequential Infection Groups

**Figure 2:**
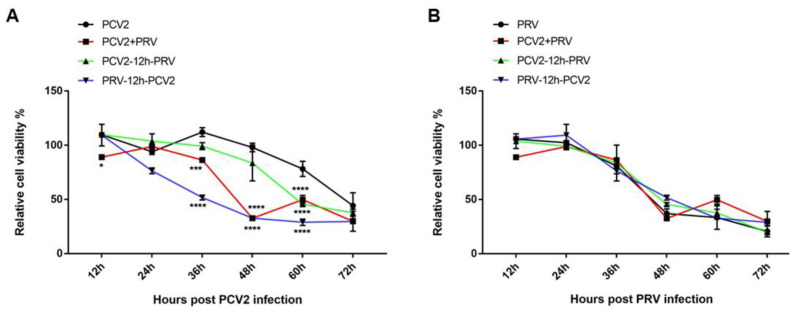
Cell viability of PCV2 (**A**) and PRV (**B**) in different infection groups. PK-15 cells were infected with different combinations of PCV2 and/or PRV for 12, 24, 36, 48, 60, and 72 h, and then the cell viability was evaluated by CCK8 at indicated hours after the indicated virus infection. *, *p*-value < 0.05; ***, *p*-value < 0.001; ****, *p*-value < 0.0001.

To evaluate the cell viability in the different infection groups, PK-15 cells were single- (PCV2 or PRV group), sequential- (groups PCV2-12h-PRV and PRV-12h-PCV2), or co-infected (PCV2+PRV group) with PCV2 and/or PRV for 12, 24, 36, 48, 60, and 72 h, followed by CCK8 assay. As shown in Figure 2A, cell viabilities were significantly decreased in one sequential infection group (PRV-12h-PCV2) and coinfection group compared with PCV2 infection alone at 36 to 60 hpi. However, there was no significant difference between the PCV2 alone group and sequential infection group PCV2-12h-PRV before 60 hpi. Moreover, the results of Figure 2B showed that sequential infection and coinfection have no significant difference in cell viabilities compared with PRV infection alone. These results indicate that PRV is the main factor for cell viability during the coinfection. In addition, the influence of the coinfection group on cell viability was similar to that of sequential infection groups. Therefore, we chose the coinfection group in the following study. 

### 3.3. Transcriptomic Analysis Revealed Systemic Alterations of Gene Expressions in Cells Single- or Co-Infected with PCV2 and PRV

**Figure 3:**
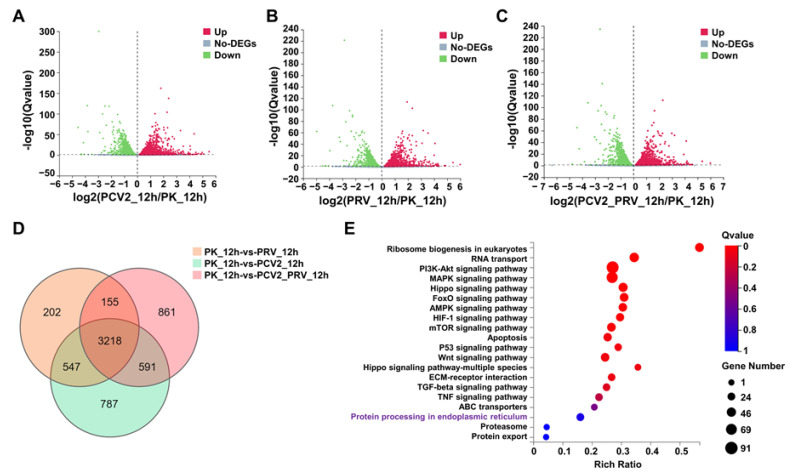

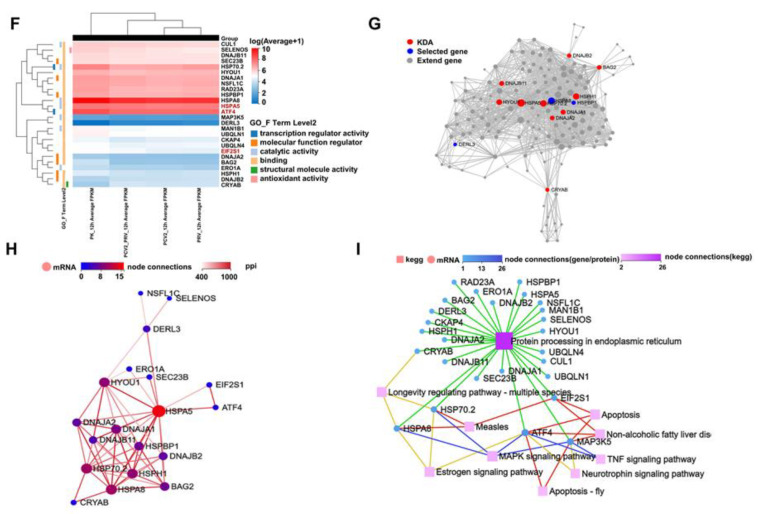
Results of transcriptome analysis. (**A**–**C**) Volcano plot of DEGs in PCV2 (**A**), PRV (**B**), and PCV2+PRV (**C**) groups compared with that of the control group (PK-15) at 12 hpi. Significant DEGs were highlighted in green (downregulated DEGs, *p* < 0.05) and red dots (upregulated DEGs, *p* < 0.05). (**D**) Venn diagram of numbers of DEGs between the control group (PK-15 cells) and different infection groups at 12 hpi. (**E**) KEGG pathway enrichment. Circles indicate the numbers of enriched genes and colors mean the Q value. Q-value < 0.05 was a significant difference. (**F**) Heatmap and one-dimensional hierarchical clustering of DEGs. (**G**) Biological networks analysis of DEGs using key driver analysis (KDA). (**H**) Biological network analysis of DEGs using protein–protein interaction network (PPI network). (**I**) Biological network analysis of DEGs using KEGG.

Transcriptomic RNA-seq analysis was performed on cells single- or co-infected with PCV2 and PRV for 12 h. Compared with the control group (PK-15), a total of 5143 differentially expressed genes (DEGs) were identified in PCV2-infected cells (2399 upregulated genes and 2744 downregulated genes), a total of 4122 DEGs were identified in PRV-infected cells (1970 upregulated genes and 2152 downregulated genes), and a total of 4825 DEGs were identified in PCV2 and PRV co-infected cells (2199 upregulated genes and 2626 downregulated genes) (Figure 3A–C). Furthermore, a total of 3218 shared DEGs were identified in three infection groups via a Venn diagram (Figure 3D). 

KEGG enrichment analysis of 3218 DEGs showed that the top 20 pathways included Ribosome biogenesis in eukaryotes, RNA transport, PI3K-Akt signaling pathway, MAPK signaling pathway, Hippo signaling pathway, Foxo signaling pathway, AMPK signaling pathway, HIF-1 signaling pathway, mTOR signaling pathway, Apoptosis, p53 signaling pathway, Wnt signaling pathway, Hippo signaling pathway-multiple species, ECM-receptor interaction, TGF-beta signaling pathway, TNF signaling pathway, ABC transporters, Protein processing in the endoplasmic reticulum (ER), Proteasome, and Protein export (Figure 3E). 

Furthermore, 26 of 3218 DEGs are related to protein processing in the endoplasmic reticulum in KEGG enrichment (Figure 3E), including HSPA5 (glucose-regulated protein 78, GRP78, also named binding immunoglobulin protein, BiP), EIF2S1 (eIF2α), and ATF4, which are associated with endoplasmic reticulum stress (ERS)-related pathways (Figure 3F). The results of KDA showed that 10 genes, including HSPA5, HSP70.2, HSP1, DNAJB11, DNAJA1, DNAJA2, DNAJB2, HYOU1, BAG2, and CRYAB, were the key related genes to the 26 DEGs in Protein processing in endoplasmic reticulum enrichment (Figure 3G). Moreover, the results of protein–protein interaction network (PPI network) analysis showed that there were direct interactions between 10 KDA genes and 26 DEGs, among which HSPA5 was a key protein, which interacted with several proteins, including ERO1A (ERO1α), DNAJA2, HSPA8, HSPH1, eIF2α, and ATF4 (Figure 3H). Moreover, biological network analysis of the 26 DEGs using KEGG assay showed that protein processing in the ER was associated with EIF2S1 (eIF2α), ATF4, and apoptosis pathway (Figure 3I).

These results indicate that infection of PCV2 and PRV alone or simultaneously could activate ERS. Therefore, the influence of PCV2 and PRV coinfection on ER activation, especially the role of GRP78 (HSPA5), eIF2α, and ATF4 in the infection, will be verified in the follow-up research.

### 3.4. PCV2 and PRV Increased GRP78 Expression during Single-Infection and Coinfection

**Figure 4:**
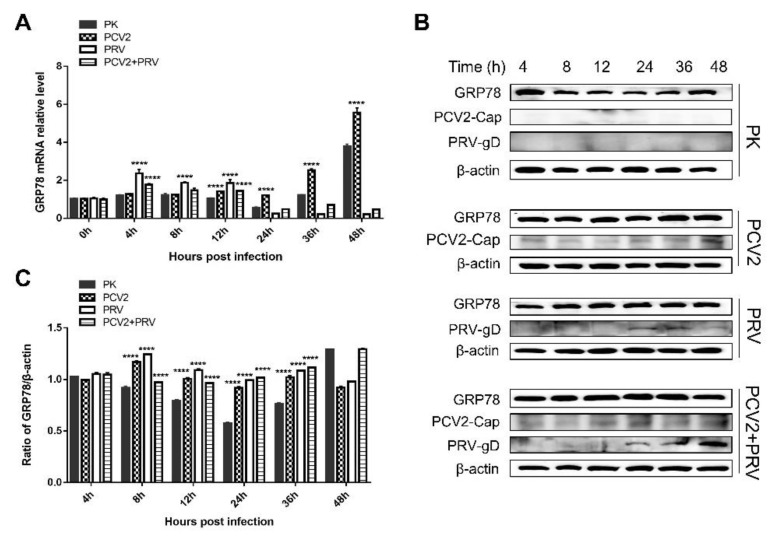
GRP78 is upregulated during PCV2 and/or PRV single- and coinfection. PK-15 cells (PK) were single- or co-infected with PCV2 and/or PRV for 4, 8, 12, 24, 36, and 48 h. The expression levels of GRP78 were assessed by real-time PCR (**A**) and Western blotting (**B**,**C**). ****, *p*-value < 0.0001. β-actin was used as a control. The relative amount of GRP78 to β-actin was quantified by densitometry analysis using ImageJ software (**C**). GRP78 (Affinity, Jiangsu, China, 1:2000), PRV-gD (Lvdu, Shandong, China, 1:500), PCV2-cap [31], and β-actin (Proteintech, Wuhan, Hubei, China, 1:10,000) were used as primary antibodies, respectively. Unprocessed original images can be found in Supplementary Figure S1.

ER chaperones are important molecules involved in the ERS, among which two critical chaperone systems, calnexin/calreticulin, and GRP78/GRP94, have been widely studied for their roles in viral infection [25,26,27,28,29,30]. Therefore, we monitored the expression of GRP78 via real-time PCR and Western blotting. The result showed that the expression levels of GRP78 were upregulated in PCV2 single infected cells at 12 to 48 hpi, and were upregulated in PRV single infected at 4 to 12 hpi compared with that of the control group (Figure 4A). The level of GRP78 was upregulated in PCV2 and PRV coinfection group at 4 and 12 hpi. These results were further confirmed by Western blotting, which showed that the levels of GRP78 were enhanced at 8 to 36 hpi in the virus single-infection and coinfection groups (Figure 4B,C). These results suggest that PCV2 and PRV increased GRP78 expression during single-infection and coinfection, which may activate the ERS. 

### 3.5. PCV2 and PRV Infection Does Not Affect the Activation of the ATF6 Pathway

**Figure 5:**
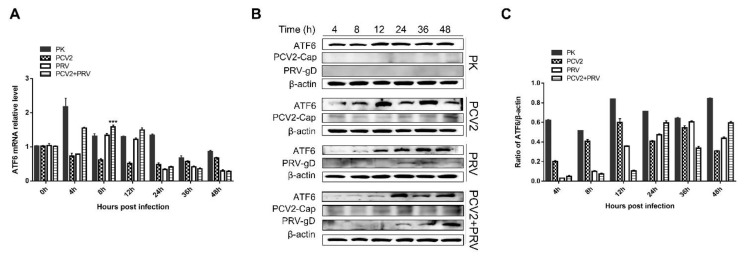
ATF6 pathway was inactivated during PCV2 and PRV single- and coinfection. PK-15 cells were single- or co-infected with PCV2 and/or PRV for 4, 8, 12, 24, 36, and 48 h. The expression levels of ATF6 were assessed by real-time PCR (**A**) and Western blotting (**B**). β-actin was used as a control. The relative amount of target protein to β-actin was quantified by densitometry analysis using ImageJ software (**C**). ***, *p*-value < 0.001. ATF6 (Wanleibio, Shenyang, China, 1:1000), PRV-gD (Lvdu, Shandong, China, 1:500), PCV2-cap [31], and β-actin (Proteintech, Wuhan, Hubei, China, 1:10,000) were used as primary antibodies, respectively. Unprocessed original images can be found in Supplementary Figure S2.

GRP78 can directly interact with three ERS sensors, ATF6, IRE1, and PERK, to regulate the UPR signaling pathways, and therefore activate ERS [25]. To clarify the downstream molecule of GRP78 during the PCV2 and PRV infection, expressions of ATF6 were evaluated. The results showed that the copies of the *atf6* gene were upregulated in the PCV2+PRV co-infected group at 8 hpi (Figure 5A), while no significant increase of ATF6 was observed in protein levels (Figure 5B,C). These results indicate that PCV2 and PRV infection alone or coinfection does not activate the ATF6 pathway.

### 3.6. PRV Infection and PCV2+PRV Coinfection Activated IRE1 Pathway

IRE1α is an ER-resident transmembrane protein with a cytosolic RNase domain [32]. Upon activation, IRE1α dissociates from the GRP78 complex and is phosphorylated, thus initiating the unconventional cytoplasmic splicing of *xbp1* mRNA [30,32]. The spliced *xbp1* mRNA encodes a transcription factor, which upregulates ER-related target protein, such as ER-degradation enhancing α-mannosidase-like protein (EDEM) [30,32]. 

**Figure 6:**
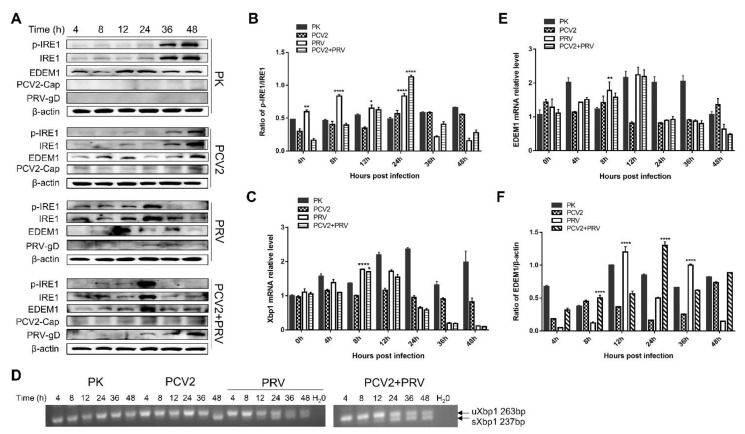
IRE1 pathway was activated in PRV single- and coinfection groups. PK-15 cells were single- or co-infected with PCV2 and/or PRV for 4, 8, 12, 24, 36, and 48 h. *, *p*-value < 0.05; **, *p*-value < 0.01; ****, *p*-value < 0.0001. (**A**) Western blotting of p-IRE1, IRE1, EDEM1, and viral proteins PCV2 Cap and PRV gD. β-actin was used as a control. (**B**) The ratio of p-IRE1α and IRE1α. Quantification of the bands corresponding to the p-IRE1α and IRE1α by densitometry was normalized to β-actin. (**C**) *xbp*1 mRNA levels quantified by real-time RT-PCR. (**D**) Splicing of *xbp*1 mRNA. Spliced *xbp*1, s*xb*p1; unspliced *xbp*1, u*xbp*1. (**E**) EDEM1 mRNA levels were quantified by real-time RT-PCR. (**F**) The relative amount of EDEM1 to β-actin was quantified by densitometry analysis using the ImageJ software. Phospho-IRE1 (Ser724) (Affinity, Jiangsu, China, 1:1000), IRE1 (Affinity, Jiangsu, China, 1:1000), EDEM1 (Affinity, Jiangsu, China, 1:2000), PRV-gD (Lvdu, Shandong, China, 1:500), PCV2-cap [31] and β-actin (Proteintech, Wuhan, Hubei, China, 1:10,000) were used as primary antibody, respectively. Unprocessed original images can be found in Supplementary Figures S3 and S4.

To elucidate the role of the IRE1 pathway in PCV2 and/or PRV infection, expressions of IRE1α, EDEM1, as well as the splicing of *xbp1* mRNA were examined. The results showed that phosphorylated IRE1α (p-IRE1) was increased in the PRV alone group from 4 to 24 hpi, and increased in the co-infected group at 24 hpi compared with that of the control group (PK-15) (Figure 6A,B), suggesting that the IRE1 pathway was activated during PRV infection and PCV2+PRV coinfection. Similarly, the copies of *xbp*1 mRNA were upregulated at 8 hpi in both PRV alone and co-infected groups (Figure 6C). Furthermore, the spliced *xbp*1 (s*xbp*1, 237 bp) and unspliced *xbp*1 (*uxbp1*, 263 bp) were detected in both PRV alone and co-infected groups from 12 to 48 hpi (Figure 6D). Moreover, the copies of *EDEM*1 mRNA were upregulated in PRV alone group at 8 hpi, and a slight increase of *EDEM*1 mRNA can be detected in the co-infected group at 8 hpi (Figure 6E). Levels of EDEM1 proteins were enhanced in the PRV group at 12 and 36 hpi, and in the co-infected group at 8 and 24 hpi (Figure 6F). Notably, levels of p-IRE1/IRE1, EDEM1, and spliced *xbp*1 were not upregulated in the PCV2 single infected group. Therefore, these results suggest that the PRV infection activates the IRE1 pathway, while PCV2 has no obvious effect on the IRE1 pathway activation.

### 3.7. PCV2 and PRV Single-Infection and Coinfection Activated the UPR via the PERK-eIF2a-ATF4-CHOP Pathway 

**Figure 7:**
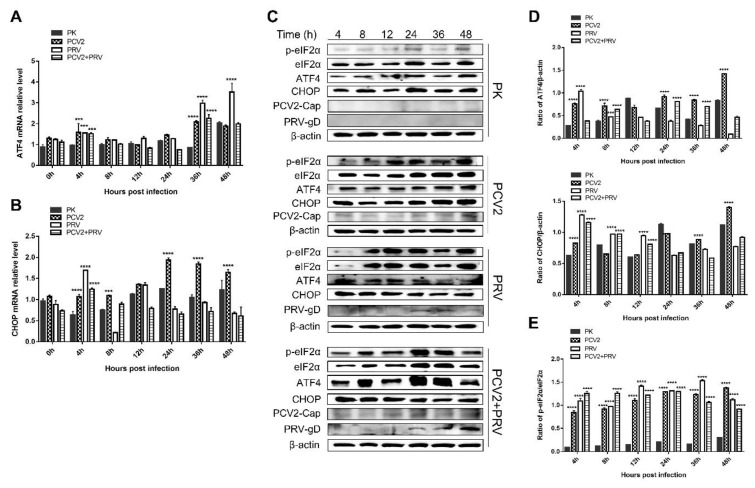
The expression levels of eIF2α, ATF4, and CHOP were upregulated during PCV2 and PRV single- and coinfection. PK-15 cells were single- or co-infected with PCV2 and/or PRV for 4, 8, 12, 24, 36, and 48 h. *p*-value < 0.01; ***, *p*-value < 0.001; ****, *p*-value < 0.0001. (**A**) ATF4 mRNA levels were quantified by real-time RT-PCR. (**B**) CHOP mRNA levels quantified by real-time RT-PCR. (**C**) Western blotting of p-eIF2α, eIF2α, ATF4, and CHOP, and viral proteins PCV2 Cap and PRV gD. β-actin was used as a control. (**D**) The relative amount of ATF4 and CHOP to β-actin was quantified by densitometry analysis using ImageJ. (**E**) The ratio of p-eIF2α and eIF2α. Quantification of the bands corresponding to the p-eIF2α and eIF2α by densitometry was normalized to β-actin. Phospho-eIF2 alpha (Ser51) (Affinity, Jiangsu, China, 1:2000), eIF2 alpha (Affinity, Jiangsu, China, 1:2000), ATF4 (Affinity, Jiangsu, China, 1:2000), DDIT3/CHOP (Affinity, Jiangsu, China, 1:2000), PRV-gD (Lvdu, Shandong, China, 1:500), PCV2-cap [31] and β-actin (Proteintech, Wuhan, Hubei, China, 1:10,000) were used as primary antibody, respectively. Unprocessed original images can be found in Supplementary Figure S5.

**Figure 8:**
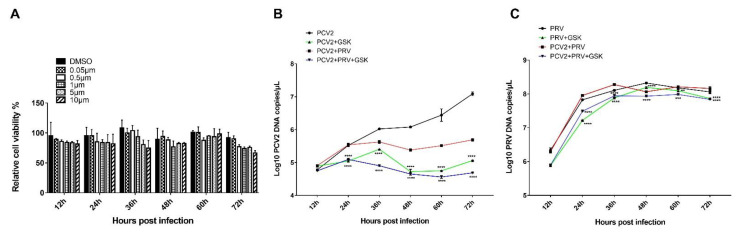
PCV2 and PRV coinfection caused PERK-mediated ERS and thus enhanced viral replication. (**A**) Effect of PERK inhibitor GSK2656157 on cell viability. PK-15 cells were treated with PERK inhibitor GSK2656157 (0.05, 0.5, 1, 5, and 10 μM) for 12, 24, 36, 48, and 72 h, followed by evaluating the cell viability via CCK8 assay. (**B**,**C**) Growth curves of PCV2 (**B**) and PRV (**C**) in PERK inhibitor-treated cells. PK-15 cells were treated with 1 μM GSK2656157, followed by single- or coinfection with PCV2 and/or PRV for 12, 24, 36, 48, 60, and 72 h. The copies of viral genes were assessed by real-time PCR. **, *p*-value < 0.01; ***, *p*-value < 0.001; ****, *p*-value < 0.0001.

PERK belongs to the type I transmembrane protein of ER. Under ERS, PERK phosphorylates itself to form a dimer, and then phosphorylates eIF2α, which inhibits the translation of protein mRNA, reduces the entry of newly synthesized protein into ER, enhances the translation of ATF4, and induces the expression of pro-apoptosis protein C/EBP homologous protein (CHOP) [15,26,30]. It was reported that PCV2 infection selectively activates the PERK pathway via the PERK-eIF2α-ATF4-CHOP axis [16,33]. The replicase (Rep) and capsid (Cap) proteins of PCV2 can induce ERS by increasing the phosphorylation of PERK and then activating the eIF2α-ATF4-CHOP axis [33]. The viral Cap significantly decreased anti-apoptotic B-cell lymphoma-2 (Bcl-2) and increased caspase-3 cleavage [33]. PCV2 can also reduce HMGB1 in the nuclei by regulating the PERK-ERO1α axis of ER, thus enhancing the viral replication [15]. Therefore, expressions of three downstream molecules of PERK, including eIF2α, ATF4, and CHOP, were evaluated. As shown in Figure 7A, copies of the *ATF* gene were enhanced in all the virus infection groups at 4 and 36 hpi compared with that of the control group (PK-15). Copies of the *chop* gene were enhanced in all the virus infection groups at 4 hpi compared with that of the control group (Figure 7B). Furthermore, copies of the *chop* gene were also enhanced in the PCV2 single infection group at 8, 24, 36, and 48 hpi, which was consistent with the results reported previously [16]. To confirm these results, Western blotting was performed to examine p-eIF2α, eIF2α, ATF4, and CHOP. The results of Figure 7C,D demonstrated that levels of ATF4 were increased at 4–8 hpi and 24–48 hpi in the PCV2 single infection group, while increased at 4–8 hpi in PRV single infection group. The coinfection of PCV2 and PRV enhanced the ATF4 at 8, 24, and 36 hpi. Meanwhile, levels of CHOP proteins were increased at 4, and 36–48 hpi in the PCV2 single infection group, whereas the levels of CHOP were enhanced at 4 to 12 hpi in the PRV single- and co-infected groups (Figure 7C,D). Moreover, ratios of p-eIF2α and eIF2α were significantly enhanced in all the infected groups at 4 to 48 hpi compared with the control group (Figure 7C,E). Furthermore, PERK inhibitor GSK2656157 (Selleckchem, Houston, TX, USA) was used to further confirm the above result. As shown in Figure 8A, PERK inhibitor GSK2656157 has no obvious effect on cell viability. The virus infection was inhibited significantly by the inhibitor at 24, 36, 48, 60, and 72 hpi (Figure 8B,C), suggesting that PCV2 and PRV infection was enhanced by the activation of the PERK pathway. 

These results indicate that the PERK-eIF2α-ATF4-CHOP axis was activated during PCV2 and PRV single- and coinfection, thus the UPR was promoted. 

## 4. Discussion

In the present study, we found that PRV infection inhibits the proliferation of PCV2, and the most obvious period of inhibition was at 36 to 72 hpi (Figure 1A). PCV2 infection enhances the proliferation of PRV, especially in the middle stage of the infection (Figure 1B). During coinfection, PRV approached the peak of proliferation in 24 h, while the proliferation rate of PCV2 was slower than that of PRV (Figure 1). PRV is the main factor for cell viability during the coinfection (Figure 2).

As we all know, virus infection and replication need cellular replication and translation systems. Both PCV2 and PRV are virulent; however, PRV infection causes CPE, while PCV2 infection cannot. PCV2 is an extremely slow-growing virus, and the virus titer produced in cell culture is very low [34,35]. The life cycle of PRV is about 4–5 hpi (viral progeny can be examined within 4–5 hpi) [36], and that of PCV2 is about 24–36 hpi [34,35]. Therefore, PRV is more virulent than PCV2, thus inhibiting PCV2 infection due to CPE caused by PRV. Moreover, virion host shutoff (vhs) protein encoded by viral *UL41* gene is a conserved protein in *Alphaherpesviruses*, which exhibits ribonuclease (RNase) activity and thus degrades mRNA, causing the shutdown of protein synthesis in host cells [36,37,38]. Meanwhile, the nuclease activity of *Alphaherpesviruses* vhs (HSV and PRV) can be stimulated by intracellular translation initiation factors, including eIF4A, eIF4B, and eIF4H [38,39,40], resulting in mRNA attenuation. Furthermore, the PRV UL41 protein preferentially targets the capped 3′-end of the internal ribosome entry site (IRES), but also cleaves downstream of the IRES region, suggesting the 5′ to 3′ RNase activity of PRV UL41 [38]. PCV2 induces immunosuppression, which is also beneficial for PRV infection. These are possible reasons why the copies of PCV2 decreased in the sequential- and co-infected groups compared with that of the PCV2 infection alone. These results further indicate that PRV plays a dominant role in the PCV2 and PRV coinfection. The exact mechanism of PRV inhibiting PCV2 remains to be studied, which is in progress in our lab. In addition, the influence of the coinfection group on virus infection was similar to that of sequential infection groups. Therefore, we chose the coinfection group (PCV2+PRV group) in the following study. 

ER is an important organelle for virus infection and replication [27,30]. During infection, viral proteins are synthesized and processed in the ER, which may lead to the accumulation of unfolded and misfolded proteins in the ER lumen of the infected cells, and then induce ERS and UPR [27,30]. UPR consists of three signaling pathways, i.e., PERK, IRE1α, and ATF6 pathways, that coordinate the adaptive response to ERS [27,30]. Furthermore, GRP78 can directly interact with three ERS sensors, ATF6, IRE1, and PERK, to regulate the UPR signaling pathways [25]. It was reported that PCV2 or PRV infection can induce ERS and UPR [15,16,17,18,19,20,21,22]. PCV2 plays a dominant role in the coinfection of PCV2 and CSFV [41]. However, there is no report on the ERS and UPR pathways caused by PCV2 and PRV coinfection. The results of the transcriptomic analysis showed that PCV2 and PRV coinfection activates ERS (Figure 3D,E), and the ERS pathway-related proteins, GRP78, eIF2α, and ATF4, were enriched (Figure 3F–H). 

GRP78 is an ER chaperone, which can translocate from the ER to the plasma membrane, and act as an ERS sensor for virus infection, cancer, and other disorders in response to cellular stress [30,42]. Under normal conditions, GRP78 binds to transmembrane UPR sensors, IRE1, PERK, and ATF6, keeping them in inactive conformation [30,42,43]. During ER stress, GRP78 dissociates from the complex, which leads to the combination of unfolded or misfolded proteins and UPR sensors, and thus activates IRE1α, PERK, and/or ATF6 pathways [30,42]. We found in this study that PCV2 and PRV increased GRP78 expression during single-infection and coinfection (Figure 4). PRV alone infection and PCV2+PRV coinfection activated the IRE1 pathway (Figure 6), which is consistent with a previous report by Yang et al. [22], whereas the IRE pathway was not induced in the PCV2 single infected group (Figure 6), suggesting a dominant role of PRV in the activation of IRE1 pathway. Moreover, the PERK-eIF2α-ATF4-CHOP axis was activated during PCV2 and PRV single- and coinfection, thus the UPR was promoted (Figure 7 and Figure 8). 

**Figure 9:**
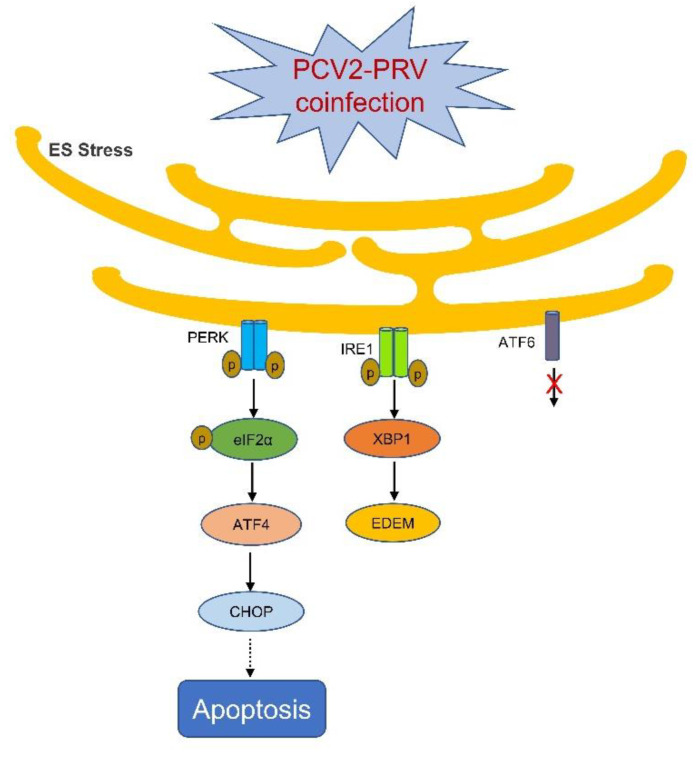
Schematic diagram of PCV2 and PRV coinfection on the activation of ERS. Arrow, confirmed; dashed arrow, needs to be clarified; ×, unactivated pathway.

It was reported that the PERK-ATF4-CHOP axis is one of the key pathways in ERS-related apoptosis and autophagy [44,45]. CHOP is a necessary transcription factor for ERS-mediated cell death, and a critical signal to modulate the expression of Bcl-2 family members [46]. PCV2 infection selectively activates the PERK pathway via the PERK-eIF2α-ATF4-CHOP axis [16,33]. The replicase (Rep) and capsid (Cap) proteins of PCV2 can induce ERS by increasing the phosphorylation of PERK and then activating the eIF2α-ATF4-CHOP axis [33]. The viral Cap significantly decreased anti-apoptotic B-cell lymphoma-2 (Bcl-2) and increased caspase-3 cleavage [33]. PCV2 also can reduce HMGB1 in the nuclei by regulating the PERK-ERO1α axis of ER, thus enhancing the viral replication [15]. Therefore, ERS can be induced during PCV2 and PRV single- and coinfections (Figure 9). Infection with PCV2 mainly activates the PERK pathway, while infection with PRV can activate both PERK and IRE1 pathways. When PCV2 and PRV are co-infected, the PERK and IRE1 pathways can be activated simultaneously, whereas PRV plays a dominant role in the coinfection. We and other groups have found that coinfection of PCV2 and PRV ranged from 1.19% to 18.75% in the field [11,47], and is responsible for porcine respiratory disease complex (PRDC) and/or other diseases [1]. Therefore, PRV infection should be the primary target to prevent and treat the diseases caused by the coinfection of PCV2 and PRV.

## 5. Conclusions

In this study, we found that PRV played a dominant role in the sequential infection and coinfection of PCV2 and PRV. PCV2 and PRV coinfection induced the ERS activation via the PERK-eIF2α-ATF4-CHOP axis and IRE1-XBP1-EDEM pathway, thus enhancing cell apoptosis and exacerbating the diseases. 

## Data Availability

All data generated or analyzed during this study are included in this published article.

## Supplementary Materials

The following supporting information can be downloaded at: https://www.mdpi.com/article/10.3390/ijms23094479/s1.
