# Supplementary material for: PCV2 and PRV Coinfection Induces Endoplasmic Reticulum Stress via PERK-eIF2α-ATF4-CHOP and IRE1-XBP1-EDEM Pathways
Source: Int J Mol Sci. 2022 Apr 19;23(9):4479. doi: 10.3390/ijms23094479 (PMC9101680; doi:10.3390/ijms23094479)
Supplement: Supplementary file 1 [file ijms-23-04479-s001.zip › ijms-1669430-supplementary.pdf]

Supplementary Information for

PCV2 and PRV coinfection induces endoplasmic reticulum stress via PERK-eIF2α-ATF4-CHOP and IRE1-XBP1-EDEM pathways

Si Chen[#], Xue Li[#], Liying Zhang[#], Xinwei Zhang, Guoyu Niu, Lin Yang, Weilong Ji, Linzhu Ren*

*** Correspondence:** Linzhu Ren
Email: renlz@jlu.edu.cn

This PDF file includes:

Figures S1 to S5

Table S1

Supplemental figures

	PK	PCV2	PRV	PCV2+PRV
GRP78	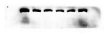		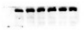	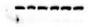
PCV2-Cap	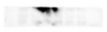	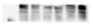		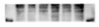
PRV-gD	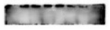		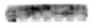	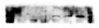

β -actin

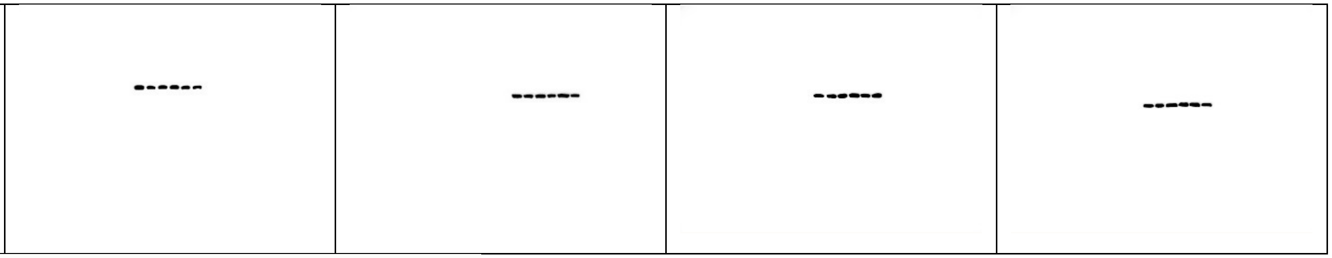

Figure S1 Original Western images used for preparing Figure 4B.

	PK	PCV2	PRV	PCV2+PRV
**ATF6**	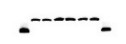	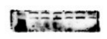	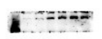	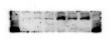
**PCV2-Cap**		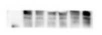		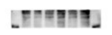
**PRV-gD**	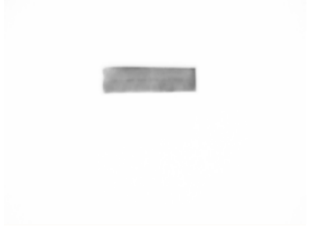		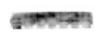	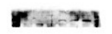
**β -actin**				

Figure S2 Original Western images used for preparing Figure 5B.

	PK	PCV2	PRV	PCV2+PRV
**p-IRE1**		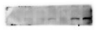	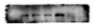	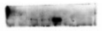
**IRE1**	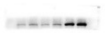	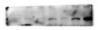	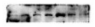	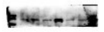
**EDEM1**	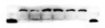	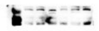	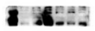	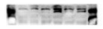

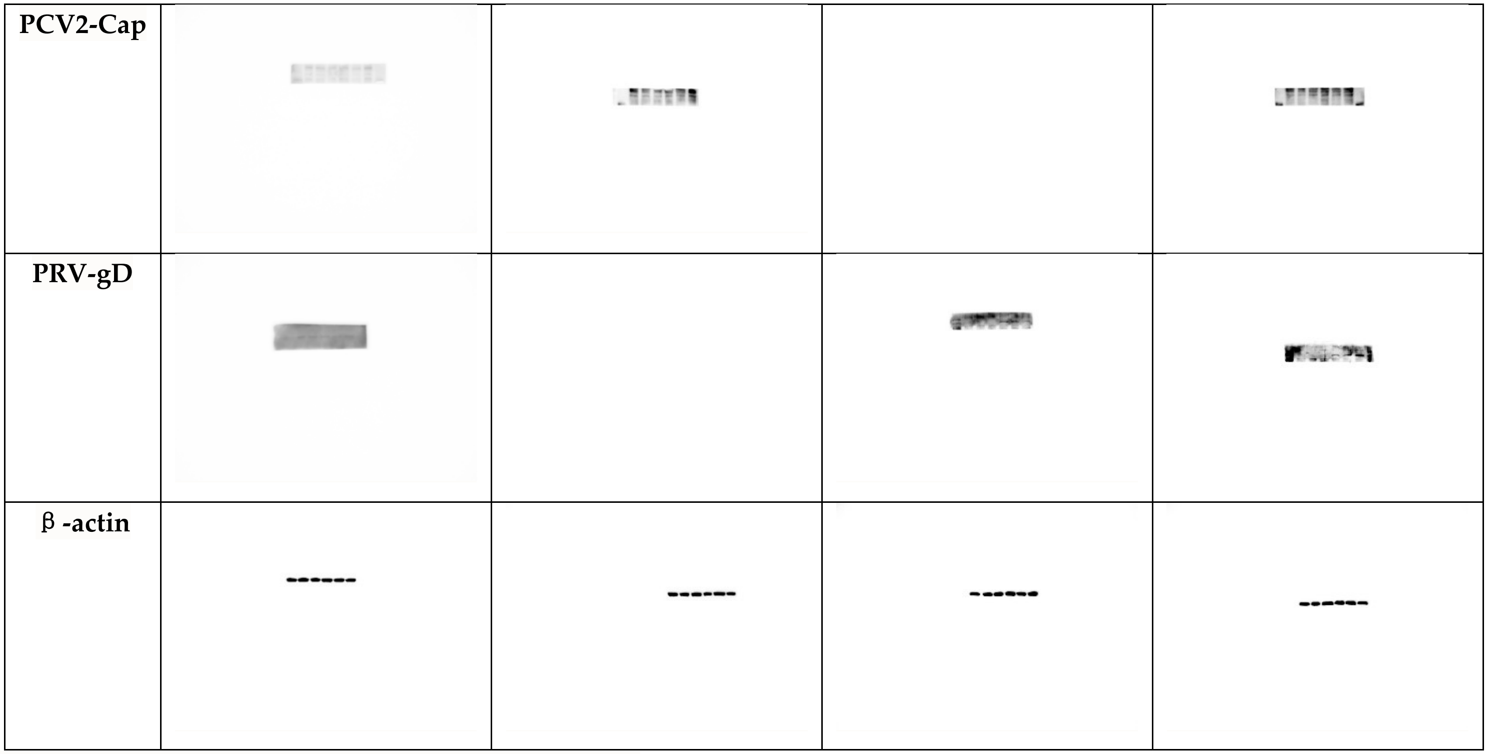

Figure S3 Original Western images used for preparing Figure 6A.

PK PCV2 PRV	PCV2+PRV
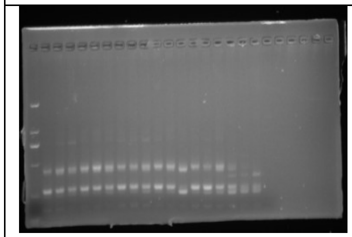	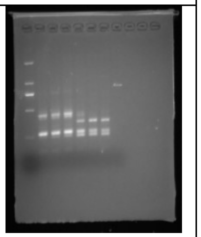

Figure S4: Original agarose gel images used for preparing Figure 6D.

	PK	PCV2	PRV	PCV2+PRV
**p-eIF2α**	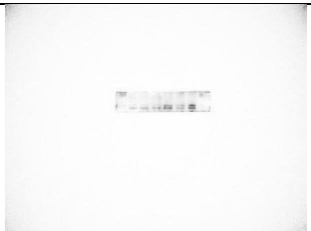	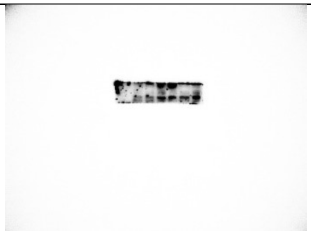	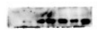	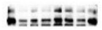
**eIF2α**			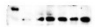	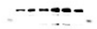
**ATF4**	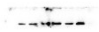	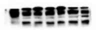	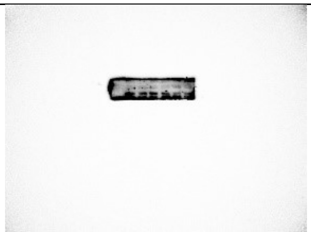	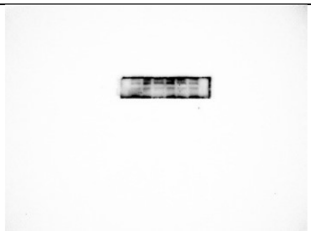

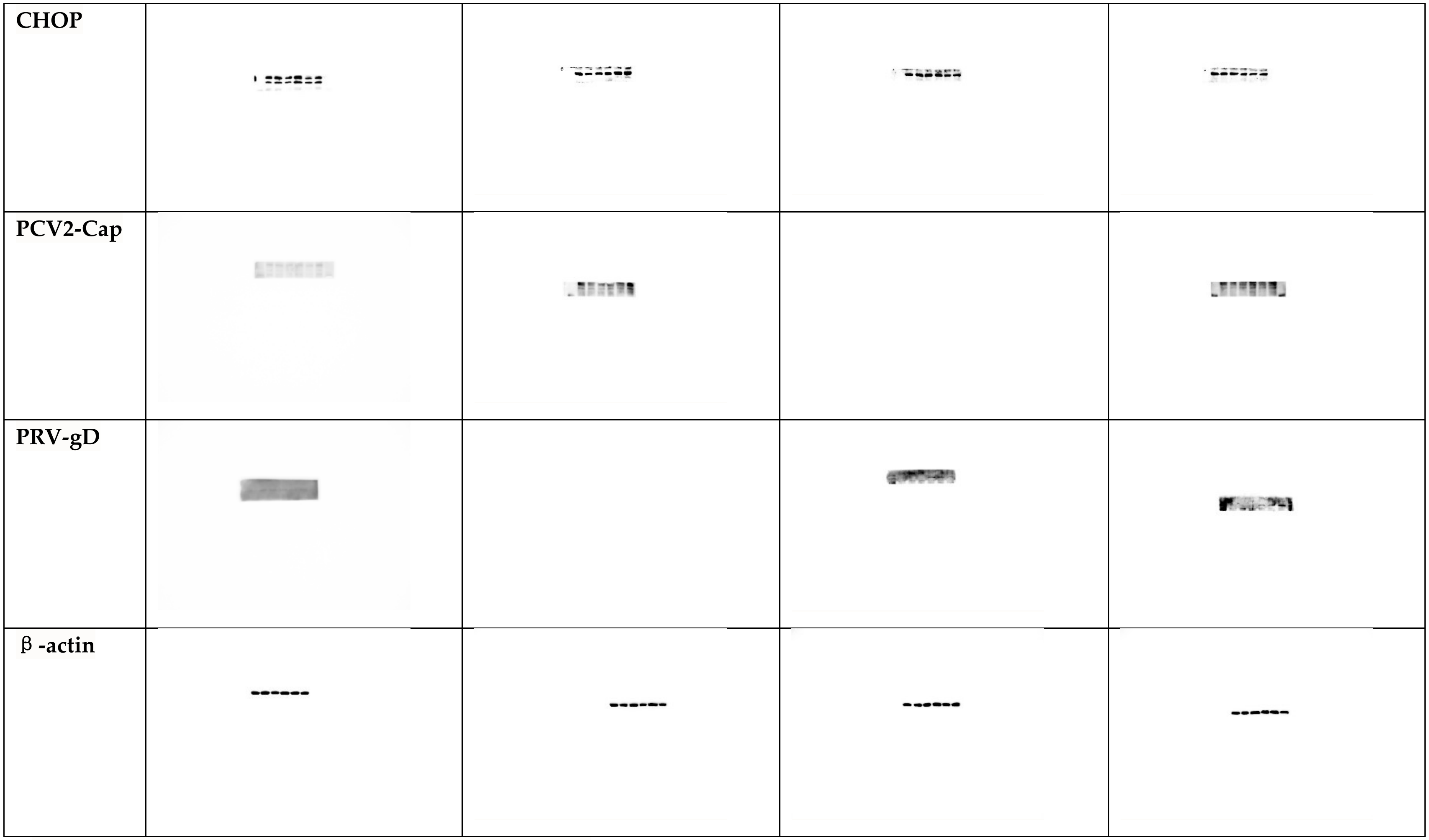

Figure S5 Original Western images used for preparing Figure 7C.

Supplemental Tables

Table S1. Primers used in this study.

Genes	Sequence (5′-3′)	GenBank ID	Size (bp)	Application
grp78	F: GTTCTTGTTGGTGGCTCTACT<br>R: ACAGCCTCATCTGGGTTTATG	XM_001927795.7	104	qPCR
atf4	F: GTGGAAATCTCGGAAGGAGATAG<br>R: AGGAGTCAGGGCTCATACA	NM_001123078.1	127	qPCR
chop	F: GAGGAGGAAGACCAAAGAAGAC<br>R: CTGTGCCACTTTCCTTTCATTC	NM_001144845.1	117	qPCR
xbp1	F: GAGACAGAGAGCCAAGCTAATG<br>R: GATACCCAGCTCTGGAATGAAG	NM_001142836.1	138	qPCR
(u/s) xbp1	F: GGCAGAGACCAAGGGGAATG<br>R: GGGTCGACTTCTGGGAGCTG	NM_001142836.1	263	PCR [16]
edem1	F: GCAATCGTGTTCCTGATGAAAG<br>R: GGGAGAGCAGGTCAAATCAA	XM_021069285.1	100	qPCR
atf6	F: ACTGGAGAGTAGGTGAGAGAAG<br>R: TTCCAAGTAGATGGGTGGATTG	XM_021089515.1	137	qPCR
gapdh	F: GCCATCACCATCTTCCAGG<br>R: TCACGCCCATCACAAACAT	NM_001206359.1	190	qPCR
PRV	F: GGTTCAACGAGGGCCAGTACCG<br>R: GCGTCAGGAATCGCATCACGT	NC_006151.1	195	qPCR
PCV2	F: TGTAGTATTCAAAGGGCACAGAGC<br>R: CGGATATACTATCAAGAAAACCAC	NC_005148.1	131	qPCR
